# The prediction of essential medicine demand using machine learning and traditional methods on EPSS Gondar hub 2018–2022 data

**DOI:** 10.1016/j.rcsop.2026.100801

**Published:** 2026-05-12

**Authors:** Tefera Minwagaw Biadigilign, Mulugojjam Jegnie Tagele, Abrham Wondimu, Wondim Ayenew

**Affiliations:** a Department of Social and Administrative Pharmacy, School of Pharmacy, College of Medicine and Health Science, Bahir Dar University, Bahir Dar, Ethiopia; b Department of Gynecology and Obstetrics, School of Medicine, College of Medicine and Health Sciences, University of Gondar, Gondar, Ethiopia; c Department of Social and Administrative Pharmacy, School of Pharmacy, College of Medicine and Health Science, University of Gondar, Gondar, Ethiopia

**Keywords:** Demand prediction, Essential medicine, Model prediction, Machine learning

## Abstract

**Introduction:**

No prior research has utilized predictive models for forecasting essential medicine demand in Ethiopia. Therefore, this study aimed to identify the most accurate predictive model for predicting essential medicine demand at public health facilities via traditional and machine learning techniques in Northwest Ethiopia.

**Methods:**

A cross-sectional study was conducted from September to December 2023 using five years of consumption data from the Ethiopian pharmaceutical supply service. The top ten essential medicines were identified based on defined daily dose utilization. Demand prediction was performed in Python using nine models, naïve, ARIMA, linear regression, support vector machine, random forest, artificial neural network, k-nearest neighbor, gradient boosting, and extreme gradient boosting. Model accuracy was evaluated using R squared, root mean squared error and mean absolute error.

**Results:**

Extreme gradient boosting was the best-performing model, achieving an R^2^ of 0.87 on both training and test sets with low RMSE and MAE values (0.18). Support vector machine and gradient boosting also performed strongly with test R^2^ of 0.87, though with higher RMSE (0.45–0.46) and MAE (0.25–0.35). The artificial neural network showed moderate performance (R^2^ ≈ 0.83) with RMSE of 0.51 and MAE of 0.31.

**Conclusions:**

The extreme gradient boosting model provided comparable model fitness with lower error estimates between the training and test datasets. Therefore, incorporating machine learning models into healthcare supply chains can significantly improve forecasting accuracy and ensure a more reliable supply of essential medicines.

## Introduction

Essential medicines (EMs) are crucial to the healthcare system and should be available at all times to prevent, treat, and cure a disease.^1^ Sustainable access to appropriate, safe, effective, and high-quality EMs through transparent processes to improve health is the primary healthcare framework of primary healthcare coverage.^2^ However, one-third of the global population lacks reliable access to EMs, and the situation is even worse in developing countries such as Africa and Asia, where more than 50% of the population lacks such access.3., 4 Owing to the significant barriers to developing and improving the effectiveness of a well-organized supply chain for EMs because of its complexity, people's access to these medicines is becoming increasingly delayed.^5^ Studies conducted in Ghana and Uganda have identified poor quantification, inappropriate medicine selection, and procurement practices as primary challenges leading to frequent stock-outs and large quantities of expired medicines in health facilities.^6^, ^7^ Despite significant efforts to address global health concerns such as HIV/AIDS, malaria, and tuberculosis over the past decade, public health facilities in developing countries still face limited access to EMs.^8^

In Ethiopia, pharmaceutical demand forecasting follows a decentralized structure, where health facilities estimate their annual medicine needs, which are then consolidated at Ethiopian Pharmaceutical Supply Service (EPSS) hubs and aggregated centrally for procurement.^9^ Despite this structure, persistent inefficiencies compromise EM availability and sustainability. National evidence shows that only 70.7% of EMs were available in warehouses and 72.4% in facility dispensaries.^10^ A systematic review further reported average stock-outs of 99.2 days and inventories containing 70.16% of EMs, fewer than WHO recommendations.^11^ In eastern Ethiopia, only 16% of surveyed medicines met the WHO 80% availability benchmark,^12^ while tracer medicines experienced average stock-outs of 30.5 days over six months, causing service disruptions due to expiries.^13^ Because Ethiopia's current demand forecasting for EMs primarily relies on manual forecasting techniques, limited technical capacity, and incomplete data, recent studies have confirmed that it is insufficient for accurately predicting public health needs. More precisely, the following issues confront Ethiopia's current EM demand prediction system:

• High forecast errors.^14^, ^15^
• Limited availability, accessibility, and visibility of reliable forecasting data.^14^, ^15^
• Shortage of advanced professional skills in demand forecasting.^14^, ^16^
• Insufficient funding and foreign currency shortages, hindering procurement.^15^
• Disruptions caused by emergencies such as COVID-19 and regional conflicts, which intensified shortages, particularly in rural and vulnerable populations.^17^
• Weak stock management practices, contributing to medicine expiries and wastage.^18^

Given Ethiopia's escalating burden of communicable and non-communicable diseases and persistent resource constraints, enhancing demand forecasting is critical to ensure equitable access, financial stewardship, and supply chain resilience.

Accurate quantification and forecasting are required for health supply chain management (SCM) to gain access to EMs.^19^ Because inadequate forecasting is frequently associated with low availability of EMs in public health facilities, increasing forecast accuracy is an urgent concern.^20^, ^21^ The lack of adequate forecasting tools is one of the primary challenges that undermines the accuracy of EM demand prediction.9., 22 Health facilities in the study area mainly rely on naïve or simple traditional forecasting methods, which are easy to apply but limited in their ability to capture trends, seasonality, or sudden shifts in demand.^23^

When demand patterns are stable and historical data is adequate, traditional forecasting techniques like regression-based approaches, moving averages, and exponential smoothing work fairly well, are transparent, and require little computational power. However, when used on complex, nonlinear, or volatile datasets, which are prevalent in the health sector, their accuracy frequently decreases.23., 24, 25 Conversely, machine learning^26^ models are made to identify hidden patterns, handle big and complicated datasets, and adjust to changing consumption patterns. Prediction accuracy can be increased by using methods like random forest (RF), support vector machines (SVM), and neural networks, particularly when the data is erratic or subject to several interrelated influences.^27^, ^28^, ^29^ In many healthcare forecasting applications, machine learning techniques have been demonstrated to perform better than traditional models, despite the fact that they are more resource-intensive and necessitate careful data preprocessing. Crucially, traditional approaches might still be helpful in some circumstances, such as when short-term, easily comprehensible forecasts are required or datasets are scarce.

A study revealed that the application of artificial intelligence (AI) and machine learning (ML) can optimize supply chain processes by enhancing demand forecasting accuracy and strengthening inventory management practices.^30^ Predictive modeling based on real-world data can therefore improve the availability of EMs by minimizing wastage, improving stock control, and ensuring timely replenishment.^31^ ML offers a flexible and data-driven way to forecast EM demand in Ethiopia, where fragmented reporting systems, supply disruptions, and irregular consumption patterns limit the reliability of traditional forecasting methods. Some countries have also recommended the integration of ML techniques into forecasting systems to enhance accuracy and optimize medicine availability.^28^, ^32^, ^33^ This suggests that the choice of predictive model, traditional or ML, should depend on the quality and quantity of available historical consumption data. However, to the best of our knowledge, no previous study in Ethiopia has applied predictive forecasting models to estimate EM demand in the study area. Therefore, this study aimed to identify the best-performing model for EM demand prediction at public health facilities in Northwest Ethiopia by evaluating multiple forecasting approaches, including both traditional and machine learning models.

## Materials and methods

### Study area, design and period

A facility-based cross-sectional study was conducted within the area of health facilities served by the EPSS Gondar Hub. The EPSS Gondar Hub is one of the 19 hubs of the EPSS and is classified under the Northwest cluster. The Hub was established in 1999. to provide services to public health facility districts found in and around Gondar. EPSS Gondar Hub serves a total of 24 public hospitals, 182 health centers, and 5 private and nongovernmental organization health facilities in the catchment area. Additionally, it provides services to 4,289,723 people in the Amhara National Regional State, who are distributed among six zones (South Gondar, Central Gondar, West Gondar, North Gondar, Wolkait Tegede Setit Humera Zones, and Gondar City Administration).^34^ The study was conducted from September 2023 to December 2023.

### Study population

All public health facilities served by the EPSS Gondar Hub were considered the source population, and all source populations that met the inclusion criteria were included in the study population. The inclusion criteria of the study were public health facilities with EMs obtained from the EPSS Gondar Hub and 5 years of complete EM consumption data between 2018 and 2022. Public health facilities that have no consumption report for two years, health facilities included in the EPSS Gondar Hub after 2018 and health facilities that faced more than a year of service interruption were excluded from the study.

### Sample size determination and sampling techniques

**Fig. 1:**
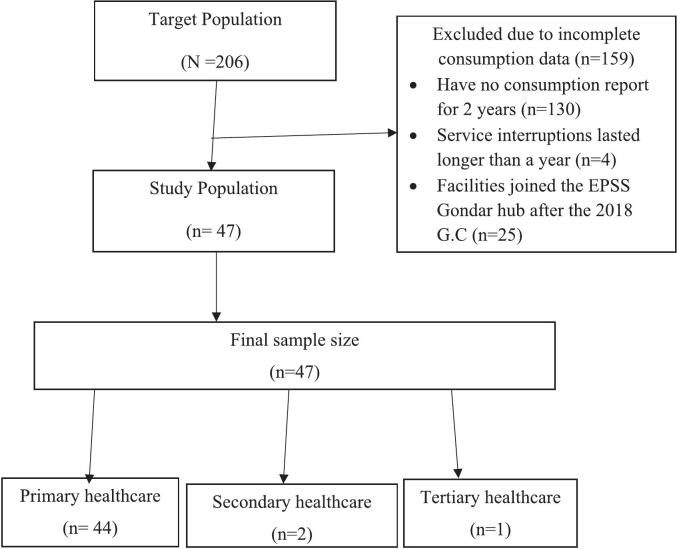
Sample selection.

In this study, all public health facilities that met the inclusion criteria were selected as the sample population. The study included 47 public health facilities, including 16 hospitals and 31 health centers. Among them, 44 were primary healthcare facilities (primary hospitals and health centers), 2 were secondary healthcare facilities (general hospitals), and 1 was a tertiary healthcare facility (comprehensive specialized hospital). Since the entire study population was selected as the sample population, this study employs a census sampling technique. (Fig. 1).

### Study variables

The quantity consumed was the dependent variable. Year, health facility, level of facility, health facility, administrative zones, level of healthcare, and types of EM were the independent variables.

### Data collection procedure and quality assurance

Two trained pharmacists collected the data from the EPSS Gondar Hub quantification and forecasting department. The health facility-reported EM consumption data were collected via a data collection checklist, which was prepared after different studies were reviewed^28^, ^35^, ^36^ and expert recommendations. The checklist included the type of EMs, the name of the health facility, the level of the facility, the level of healthcare, the year, and the amount of each EM consumed by each health facility. The collected consumption data of the health facilities were used to train and test the predictive models and were available yearly. Five years of consumption data (2018 to 2022) for EMs were collected.  Annual data were used because they were the most complete and consistently available in the EPSS database, whereas monthly or quarterly records were often missing or inconsistently reported across facilities during the study period. The principal investigators validated and reconciled the EPSS data by comparing reported consumption with stock records, and imputation for missing or inconsistent entries. Data confidentiality was strictly maintained through anonymization and secure storage.

### Data processing and analysis

Following data collection, the EPSS Gondar Hub's total health facility consumption panel structured (facility × medicine × year) data was imported into Excel for cleaning, processing, and analysis using Python and the Anaconda distribution's Jupyter Notebook. Seven factors were examined in this study: the type of EM, the amount of EM consumed, the administrative zone, the level of care, the facility level, the year, and the health facility.

After data preprocessing activities, the top 10 most highly consumed EMs were identified based on their defined daily dose (DDD) consumption, which represents the average maintenance dose per day for a drug used for its main indication in adults.^37^ DDD was selected as it standardizes medicine use across different strengths and dosage forms for reliable comparison, and the study focused on the top 10 most consumed EMs to ensure sufficient data variability and statistical robustness, thereby enhancing model reliability and practical relevance for supply chain decision-making. Additionally, it allowed forecasting models to capture more consistent and statistically sound demand patterns, increasing the accuracy of predictions.^28^, ^36^, ^38^

Consumption in DDD = Total amount of drug consumed in a given period in grams.

Standard WHO DDD in grams.

The Anatomical Therapeutic Chemical^26^ classification system of the WHO, which divides medicines into various groups on the basis of their therapeutic use and chemical characteristics, was used to classify EMs.

The target variable's distribution was first assessed using histograms and the Shapiro–Wilk test to evaluate normality. Missing values were imputed using the median, a robust measure for skewed consumption data, ensuring a complete dataset. Outliers were then identified through boxplots and *Z*-scores, and extreme values were either removed (Z > |3|) or winsorized at the 1st and 99th percentiles depending on the variable distribution. Temporal patterns were captured using the “year” variable as a continuous feature, allowing the models to learn general trends from limited annual data. Categorical variables were encoded using one-hot encoding, and all numeric features were scaled with Min-Max normalization fitted on the training set, with the same transformation applied to the test set. Multicollinearity among predictors was assessed using the variance inflation factor, with a threshold of 5; all predictors were retained as none exceeded this cutoff. Finally, descriptive statistics of the target variable (mean, median, standard deviation, min–max) were calculated, and relationships with predictors were explored using Pearson correlation analysis (*p* < 0.05). Following preprocessing, the dataset was split into training and test sets using a random 80/20 approach. Due to the limited number of annual observations, a strictly time-aware split was not feasible without substantially reducing the training data. Predictive models included linear regression, SVM, RF, artificial neural network (ANN), k-nearest neighbors (KNN), gradient boosting (GB), and extreme gradient boosting (XGB). The models were implemented as supervised regression approaches incorporating temporal features. Conventional approaches, including the naïve method and autoregressive integrated moving average (ARIMA), were also evaluated for comparison. Hyperparameter tuning for ML models was performed using grid search optimization. Model performance was assessed using the coefficient of determination (R^2^), root mean square error (RMSE), and mean absolute error^39^.^40^ Data processing was performed before model training via log transformation methods to reduce overfitting.

### Operational definitions

*Complete consumption data:* Comprehensive records detailing the type of EM, previous consumption, year, and name of the health facility, covering EM used over five years.

*Consumption data:* Quantity of EMs consumed by each health facility during the study period.

*Essential medicine:* Revolving drug fund EMs included in the Ethiopian EML and the EPSS procurement list, as well as those available at the health facilities within the study area.

*Predictive model:* The predictive model encompasses the traditional and ML techniques employed to predict the demand for EMs.

*Traditional forecasting methods:* Forecasting methods involve nonseasonal time series forecasting techniques used to predict EM demand based on historical consumption data.

*RMSE:* the average size of prediction errors, indicating how much the model's forecasts deviate from the observed values.

*MAE:* The typical absolute difference between predicted and actual consumption, providing a straightforward assessment of forecast accuracy.

*R*^*2*^*:* The proportion of variability in medicine demand that the model can explain, with higher values reflecting better predictive performance.

## Results

### Essential medicine selection

Over 500 EMs from the sixth edition of the EM list were cross-referenced with those from the fifth edition of the EM list and the first and second EPSS procurement lists for hospitals and health centers. This process identified 124 EMs, from which the top 10 most consumed were selected for analysis on the basis of their total DDD consumption. These included amoxicillin 500 mg capsules, ciprofloxacin 500 mg tablets, diclofenac sodium 50 mg tablets, doxycycline 100 mg capsules, ferrous sulfate 325 mg tablets, hydrochlorothiazide 25 mg tablets, metronidazole 250 mg capsules, omeprazole 20 mg capsules, paracetamol 500 mg tablets, and phenobarbital 100 mg tablets. These EMs were classified according to the WHO's ATC classification. The selected EMs included different therapeutic agents, such as drugs for peptic ulcer disease, antibacterial, analgesics, anticonvulsants, antihypertensive, antianemic, and antiprotozoal. (Table 1).

**Table 1:** Description of selected EMs.

ATC Code	Drug Name	Pharmacotherapeutic Group
J01CA04	Amoxicillin 500 mg	Penicillin antibiotics
J01MA02	Ciprofloxacin 500 mg	Fluoroquinolone antibiotic
M01AB05	Diclofenac Sodium 50 mg	Nonsteroidal anti-inflammatory drug
J01AA02	Doxycycline 100 mg	Tetracycline antibiotic
B03AA07	Ferrous Sulfate 325 mg	Antianemia iron preparation
C03AA03	Hydrochlorothiazide 25 mg	Thiazide diuretic antihypertensive
P01AB01	Metronidazole 250 mg	Antiprotozoal, nitroimidazole Antibiotic
A02BC01	Omeprazole 20 mg	Peptic ulcer disease, proton pump inhibitor
N02BE01	Paracetamol 500 mg	Analgesic and antipyretic
N03AA02	Phenobarbital 100 mg	Barbiturate anticonvulsant

### Consumption of essential medicines by DDD

On the basis of consumption in DDD, the four most common EMs were omeprazole 20 mg capsules (23,063,300), amoxicillin 500 mg capsules (14,002,000), phenobarbital 100 mg tablets (12,339,000), and hydrochlorothiazide 25 mg tablets (9,402,300) (Table 2).

**Table 2:** Quantity of EMs consumed in DDD.

Selected Ems	Consumption in DDD
Omeprazole 20 mg capsule	23,063,300
Amoxicillin 500 mg capsule	14,002,000
Phenobarbital 100 mg tablet	12,339,000
Hydrochlorothiazide 25 mg tablet	9,402,300
Ciprofloxacin 500 mg tablet	8,696,500
Ferrous Sulfate 325 mg tablet	7,949,663
Doxycycline 100 mg capsule	7,466,200
Paracetamol 500 mg tablet	6,383,500
Diclofenac Sodium 50 mg tablet	6,277,500
Metronidazole 250 mg capsule	6,223,750

### Consumption of essential medicines by time

**Fig. 2:**
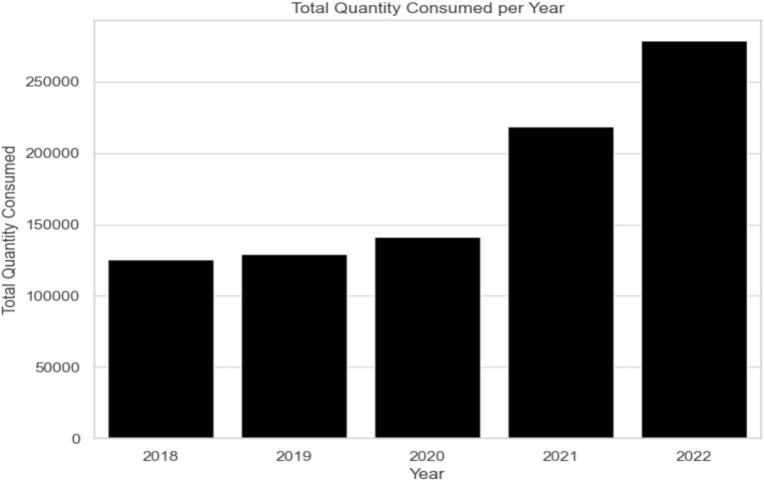
The total quantity of all selected EMs consumed over year.

As showed in figures below, the total consumption of all selected EMs by health facilities increased between 2018 and 2022 (Fig. 2).

**Fig. 3:**
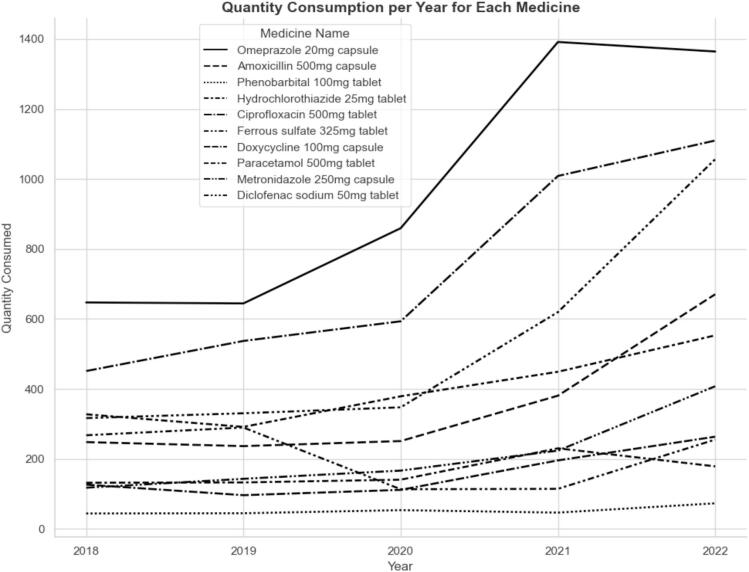
Quantity of each EMs consumed over time.

The consumption of certain EMs, including metronidazole, diclofenac, and ciprofloxacin, increased steadily between 2018 and 2022. Although the consumption of omeprazole and paracetamol in 2022 was slightly lower than that in 2021, it was still higher than that in the three preceding years. Similarly, the consumption of hydrochlorothiazide and amoxicillin increased overall, except for a dip in 2019 compared with 2018. However, ferrous sulfate and doxycycline consumption fluctuated throughout the study period. (Fig. 3).

### Application machine learning for predictive modeling

The data were divided into training and test sets to evaluate the generalizability of the predictive model to new data by training it on one set and testing it on another. The training set included 80% of the data from the datasets, whereas the test set included 20% of the consumption data. After removing 37 extreme outliers identified by *Z*-scores greater than ±3, the final dataset included 2313 observations, 1850 for training (mean = 304.21 ± 410.19) and 463 for testing (mean = 280.22 ± 357.13) (Table 3).

**Table 3:** Summary statistics of the quantity of EM consumed for the training and test sets.

Set	Variable	Count	Mean	SD.	Median	Min	Max	IQR
Train	Quantity consumed	1850	304.21	410.19	157.5	2	2863	250
Test	Quantity consumed	463	280.22	357.13	167	2	2400	231.5

### Prediction of essential medicine demand with predictive models

The demand prediction for EMs was evaluated using predictive models from diverse categories, including traditional forecasting methods, ML, ensemble methods, and deep learning approaches. To enhance the accuracy of hyperparameter-dependent ML models such as Random Forest, ANN, KNN, GB, and XGB this study employed grid search optimization for parameter tuning.

The XGB model outperforms the other predictive models. It fit 87% of the test and training datasets with a RMSE and MAE of 0.18. Comparatively, GB, SVM, and ANN fit 87%, 88%, and 84% of the training set, respectively, and both GB and SVM fit 87% of the test set, whereas the ANN fit 83%. In contrast, ARIMA fit 62% with a low root mean squared error. Linear regression fit 60% of the training dataset and 62% of the test set with a higher RMSE than XGB, GB, and SVM. The ARIMA model had a moderate RMSE (0.51) and moderate R^2^(62%), whereas the naïve model's performance was poor, with a moderate RMSE (0.55) and low R^2^(48%) (Table 4).

**Table 4:** RMSE and R^2^ results of the predictive models.

Model	Data set	RMSE	R-Squared	MAE
XGB	Train	0.17907	0.87165	0.18
	Test	0.17964	0.86961	0.18
Linear Regression	Train	0.77	0.60	0.58
	Test	0.77	0.62	0.56
ANN	Train	0.49	0.84	0.27
	Test	0.51	0.83	0.31
SVM	Train	0.42	0.88	0.22
	Test	0.45	0.87	0.25
Random Forest	Train	33.04	0.93	23.79
	Test	74.64	0.67	61.18
Gradient Boosting	Train	0.45	0.87	0.34
	Test	0.46	0.87	0.35
KNN	Train	0.50213	0.83271	0.35
	Test	0.65229	0.72686	0.46
Naïve	Train	0.5669	0.4808	0.45
	Test	0.5495	0.4755	0.44
ARIMA	Train	0.45	0.65	0.41
	Test	0.51	0.62	0.46

**Fig. 4:**
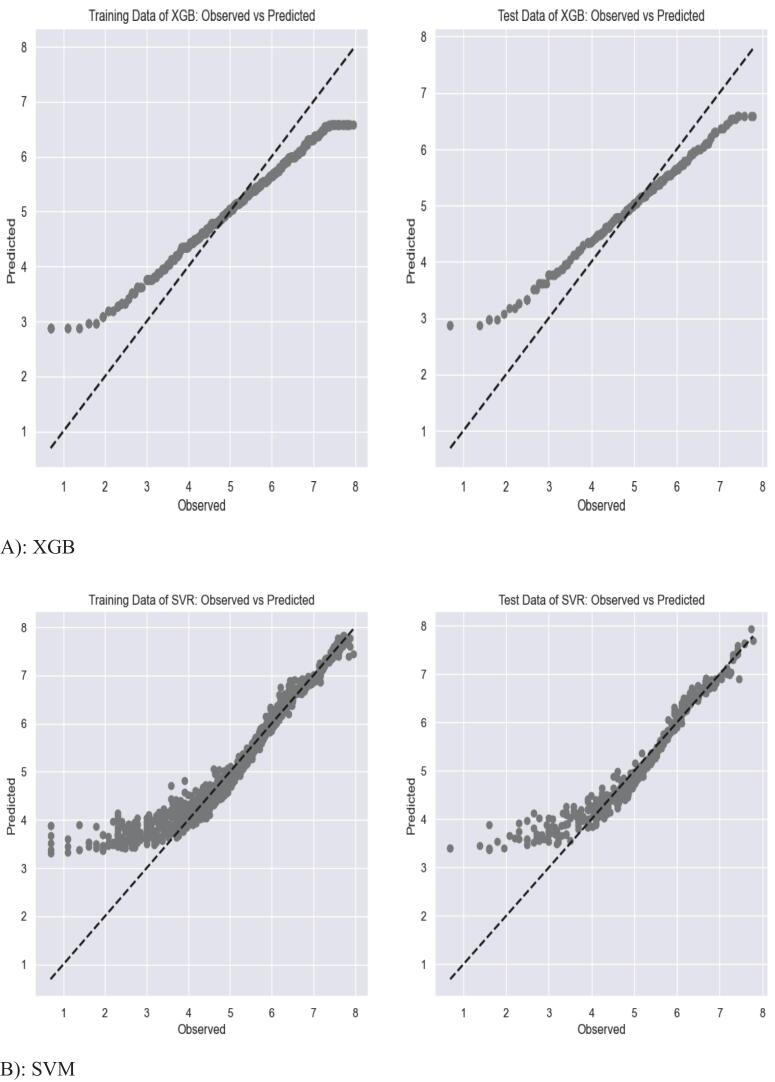

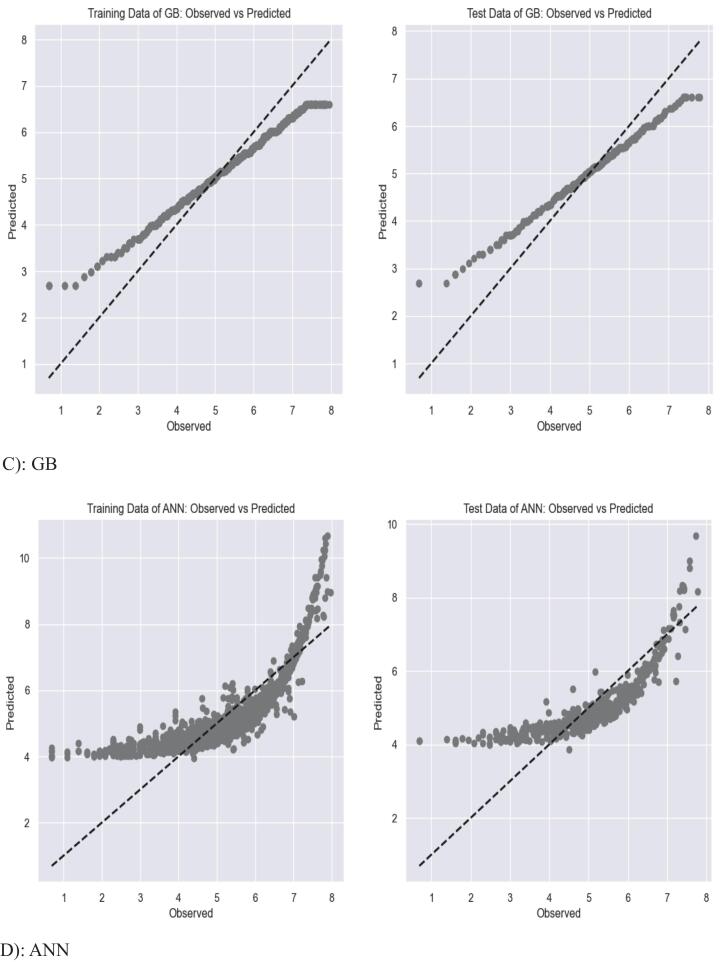
Accuracy of the top 4 predictive models on the training and test sets.

The graphical representation for the accuracy of the predictive models is presented in Fig. 4 below. Owing to the challenge of including performance accuracy graphs for all the models, only the top four most accurate prediction models for EM demand on the training and testing sets are shown below. (Fig. 4).

## Discussion

The evaluated models demonstrated notable differences in forecasting performance. Among all approaches, XGB achieved the highest predictive accuracy, with the lowest RMSE (0.18) and a high-test R^2^ (0.87). Compared with the naïve baseline model, XGB reduced prediction error by approximately 67% and improved explanatory performance by more than 39 percentage points. In practical terms, this corresponds to an estimated reduction of about 887 DDDs in forecast error per medicine per facility, highlighting the model's potential to reduce overstocking, medicine expiry, and stock-out risks in health facilities.^41^ SVM and GB also showed strong predictive performance, indicating that ML approaches are better able to capture the nonlinear and heterogeneous consumption patterns commonly observed in essential medicine demand. XGB's superior accuracy can be attributed to its ability to capture complex feature interactions and apply built-in regularization to limit overfitting. Similarly, SVM's kernel functions effectively handle overlapping patterns and noise. These strengths align well with the dataset's heterogeneous and seasonal nature, explaining why ML models outperform linear and traditional time-series methods in this context.^42^

In contrast, the naïve, ARIMA, and linear regression models demonstrated lower predictive performance. Although ARIMA performed better than the naïve and linear regression models, its accuracy remained below that of the leading ML models, likely due to the complex and nonstationary nature of medicine consumption patterns in the study setting. Linear regression showed the weakest performance, reflecting its limited ability to model nonlinear relationships.^25^ The ANN model demonstrated relatively stable performance between training and testing datasets, suggesting acceptable generalization capability and improved robustness compared with traditional approaches.

Both RF and KNN showed evidence of overfitting, although the effect was more pronounced in RF. The RF model achieved very high training performance (R^2^ = 0.93; RMSE = 33.04; MAE = 23.79) but substantially lower testing accuracy (R^2^ = 0.67; RMSE = 74.64; MAE = 61.18), yielding ΔR^2^ = 0.26, ΔRMSE = 41.6, and ΔMAE = 37.39. This may be related to the limited dataset size, high variability across medicine–facility combinations, and the tendency of tree-based models to fit noise in sparse datasets. KNN also exhibited moderate overfitting, likely due to its sensitivity to local data variation and limited subgroup observations (training: R^2^ = 0.83, RMSE = 0.50, MAE = 0.35; testing: R^2^ = 0.73, RMSE = 0.65, MAE = 0.46). These differences reflect KNN's sensitivity to local data noise and the limited data points per subgroup. Hyperparameter tuning using grid search helped balance bias and variance across all models.

From a public health perspective, accurate demand forecasting for EMs is crucial for ensuring consistent access to life-saving treatments. Poor forecasting contributes to both overstocking and stock-outs, reducing medicine availability and compromising health outcomes—especially in rural and resource-limited areas.9., 11, 12, 43 By applying advanced ML methods, this study demonstrates how data-driven forecasting can improve demand prediction and support more reliable access to EMs in Ethiopia. The superior performance of XGB, SVM, and ANN highlights the ability of ML to manage uncertainty, handle complex data patterns, and improve forecast precision compared with traditional methods such as ARIMA and naïve models.^25^, ^29^, ^31^, ^44^

Previous Ethiopian studies have largely focused on stock-out assessment and traditional quantification practices, without applying supervised predictive models for essential medicine demand forecasting. This study contributes to advancing data-driven forecasting approaches in resource-limited health systems. Consistent with global evidence, the findings suggest that ML-based forecasting can improve supply chain efficiency by reducing stock imbalances, supporting procurement planning, and improving responsiveness to fluctuations in medicine demand, including during emergencies and outbreaks.^33^, ^45^, ^46^

Successful implementation of ML-based forecasting in the health supply chain also requires strong ethical and governance considerations. Forecasting systems should support equitable allocation of medicines across facilities and avoid bias toward high-volume settings. In addition, clear data governance policies are needed to guide data quality management, access, sharing, and privacy protection. ML-generated forecasts should support, rather than replace, human decision-making, with appropriate oversight mechanisms to ensure responsible use within the public health system.

Several operational challenges may affect practical implementation. Incomplete or inconsistent data recording remains a major concern, particularly in facilities relying on manual documentation systems.^47^, ^48^ Strengthening digital reporting systems, improving data validation procedures, and ensuring consistent data entry practices are therefore essential.^49^ Limited ICT infrastructure, especially in rural settings, may also constrain implementation, although cloud-based and mobile-supported platforms could improve accessibility in low-resource environments.^50^, 51. Continuous capacity-building for supply chain professionals and health workers will also be necessary to support interpretation and practical use of ML-generated forecasts.

The current model was developed using data from facilities served by the EPSS Gondar Hub and was intended primarily for hub-level forecasting. Expanding the approach to additional EPSS hubs and incorporating program and non-revolving drug fund medicines could improve generalizability and support broader national implementation.

This study has several limitations. First, removing outliers may have excluded genuine but unusual consumption spikes linked to outbreaks or delayed stock replenishment. Second, using DDDs as a standardized metric may underrepresent medicines with variable or weight-based dosing, such as pediatric formulations. Third, restricting the dataset to the top ten most-consumed EMs and to facilities under the Gondar Hub may introduce regional and selection bias, limiting the model's applicability to lower-volume medicines and other regions. Finally, using annual data rather than more granular monthly or quarterly records may mask seasonal variations and short-term fluctuations. ARIMA's modest performance also reflects the short five-year series, underscoring the need for more frequent data collection to enhance time-series models. The use of a random data split may introduce temporal leakage, as observations from different years could appear in both training and testing sets. Future studies should consider time-aware validation approaches, such as temporal hold-out or rolling-origin validation, to improve robustness.

## Conclusion

This study found that XGB achieved the best performance for forecasting EM demand among the evaluated models in public health facilities of Northwest Ethiopia. The improved predictive accuracy of the model may help strengthen medicine availability by supporting better procurement planning, reducing stock-outs, and minimizing medicine wastage.

The findings also indicate that ML-based forecasting could be integrated into the EPSS system to support data-driven decision-making. Linking the model with LMIS dashboards may improve real-time monitoring of medicine consumption, stock status, and procurement planning. Effective implementation, however, will require adequate digital infrastructure and practical training for supply chain personnel.

Future studies should validate the model across multiple EPSS hubs using more detailed and time-sensitive datasets. Incorporating additional predictors such as lag features, disease outbreaks, seasonal variations, and supply disruptions may further improve forecasting performance and support more resilient medicine supply systems in Ethiopia.

## CRediT authorship contribution statement

**Tefera Minwagaw Biadigilign:** Writing – review & editing, Writing – original draft, Visualization, Software, Methodology, Formal analysis, Data curation, Conceptualization. **Mulugojjam Jegnie Tagele:** Writing – review & editing, Writing – original draft, Validation, Conceptualization. **Abrham Wondimu:** Writing – review & editing, Validation, Supervision, Conceptualization. **Wondim Ayenew:** Writing – review & editing, Writing – original draft, Validation, Supervision, Methodology, Conceptualization.

## Ethical approval and consent to participate

Ethical clearance was obtained from the University of Gondar, School of Pharmacy, Ethical Review Committee, with reference number SOP/662/2023. All procedures in this study were conducted in accordance with the Declaration of Helsinki. The quantification and forecasting department's EPSS hub records were reviewed with permission from our department (Issue No: S/A/P/48/2023, Date: 22/5/2023), which was formally confirmed by the hub head. The data was stored on a computer that was password-protected to ensure its confidentiality.

## Funding

This study did not receive any specific funding.

## Declaration of competing interest

The authors declare that they have no known competing financial interests or personal relationships that could have appeared to influence the work reported in this paper.

The author is an Editorial Board Member/Editor-in-Chief/Associate Editor/Guest Editor for this journal and was not involved in the editorial review or the decision to publish this article.

The authors declare the following financial interests/personal relationships which may be considered as potential competing interests:

## Data Availability

The dataset supporting this study is available from the corresponding author upon reasonable request.
