# Supplementary material for: The prediction of essential medicine demand using machine learning and traditional methods on EPSS Gondar hub 2018–2022 data
Source: Explor Res Clin Soc Pharm. 2026 May 12;23:100801. doi: 10.1016/j.rcsop.2026.100801 (PMC13197753; doi:10.1016/j.rcsop.2026.100801)
Supplement: Supplementary file 1 — Supplementary material [file mmc1.docx]

| Model | Hyperparameters | Overfitting detection | Mitigation strategy |
| --- | --- | --- | --- |
| LR | Regularization = None | Lowest RMSE on test set | Train–test error |
| RF | Max depth = 20; Max features = 7; Min samples per leaf = 2; n_estimators = 500; Random state = 42 | Train–test performance gap | Depth and leaf constraints |
| XGB | n_estimators = 10; Learning rate = 0.1; Max depth = 3; Random state = 42 | Train–test performance gap | Shallow trees, low learning rate, early stopping |
| GB | Learning rate = 0.1, Max depth = 3, Min sample split = 2, n_estimators = 200, Subsample = 0.8 | Train–test performance gap | Subsampling, shallow trees |
| ANN | Hidden layers = 3, Input dim = 7, Activation = ReLU, Optimizer = Adam, Epochs = 20, Batch size = 3, Validation split = 0.2 | Train–test loss comparison | Limited epochs, validation split, early stopping |
| SVM |  | Lowest validation RMSE during grid search | ΔR² between train and test |
| KNN | k = 5, weights = ‘uniform’, metric = ‘minkowski’ | Train–test performance gap | Balanced bias–variance trade-off |
| ARIMA | Order = (2,1,1) | Model fit comparison | Consistent order across series |

**Supplementary (S1):** Hyperparameter Tunning

***Supplementary (S2): Sample of Python commands used in the study***

#Load libraries

- import pandas as pd
- import numpy as np
- from pandas import read_excel

#load dataset

- data **=** pd**.**read_excel("SM3.xlsx")

#data description

- data['Quantity_Consumed']**.**describe()
- data**.**head()
- data**.**tail()
- data**.**columns**.**tolist()

**#Data visualazation**

- import matplotlib.pyplot as plt
- import seaborn as sns
- %matplotlib inline
- sns.set(color_codes=True)

*# Assuming you have a DataFrame called 'data' with columns 'Year' and 'Quantity_Consumed'*

- sns**.**barplot(x**=**'Year', y**=**'Quantity_Consumed', data**=**data)
- plt**.**show()
- *# Assuming you have a DataFrame called 'data' with columns 'Year', 'Level_of_Healthcare', 'Medicine_Name', and 'Quantity_Consumed'*
- sns**.**catplot(x**=**'Year', y**=**'Quantity_Consumed', hue**=**'Level_of_Healthcare', kind**=**'bar', data**=**data)
- plt**.**show()

#Missing value and filling with median

- data.isnull().sum()/100
- data["Quantity_Consumed"]=data["Quantity_Consumed"].fillna(data["Quantity_Consumed"].median())

#Identify outliers using box plots and zscore

- plt.boxplot(data['Quantity_Consumed'])
- plt.title('Box Plot of Quantity Consumed')
- plt.show()
- from scipy.stats import zscore
- data['Quantity_Consumed_zscore'] = zscore(data['Quantity_Consumed'])
- data = data[(data['Quantity_Consumed_zscore'] < 3) & (data['Quantity_Consumed_zscore'] > -3)]

#Train_test data split

- data_X = data.drop(columns=['Quantity_Consumed'])
- data_y = data['Quantity_Consumed']
- from sklearn.model_selection import train_test_split
- *# Assuming you have a dataset called 'data' and the target variable is called 'target'*
- X_train, X_test, y_train, y_test = train_test_split(data_X, data_y, test_size=0.2, random_state=42)

#Descriptive data for train and test set

- print("\nTraining Target Description:")
- print(y_train.describe())
- print("\nTesting Target Description:")
- print(y_test.describe())

#Model prediction

#Linear regression

- from sklearn.metrics import mean_squared_error, r2_score, mean_absolute_error

# Train a linear regression model

- model_lr = LinearRegression()
- model_lr.fit(X_train, y_train)

# Make predictions on the train and test sets

- y_train_pred_lr = model_lr.predict(X_train)
- y_test_pred_lr = model_lr.predict(X_test)

# Calculate RMSE, R², and MAE for the training set

- train_rmse = np.sqrt(mean_squared_error(y_train, y_train_pred_lr))
- train_r2 = r2_score(y_train, y_train_pred_lr)
- train_mae = mean_absolute_error(y_train, y_train_pred_lr)

# Calculate RMSE, R², and MAE for the test set

- test_rmse = np.sqrt(mean_squared_error(y_test, y_test_pred_lr))
- test_r2 = r2_score(y_test, y_test_pred_lr)
- test_mae = mean_absolute_error(y_test, y_test_pred_lr)

# Print results

- print(f"Train Set RMSE of LR: {train_rmse:.2f}")
- print(f"Train Set R² of LR: {train_r2:.2f}")
- print(f"Train Set MAE of LR: {train_mae:.2f}")
- print(f"Test Set RMSE of LR: {test_rmse:.2f}")
- print(f"Test Set R² of LR: {test_r2:.2f}")
- print(f"Test Set MAE of LR: {test_mae:.2f}"):

#Random Forest

- from sklearn.ensemble import RandomForestRegressor

#Identify best hyperparameters

- from sklearn.ensemble import RandomForestRegressor
- from sklearn.model_selection import GridSearchCV
- from sklearn.datasets import make_regression
- from sklearn.model_selection import train_test_split
- from sklearn.metrics import mean_squared_error
- # Generate a sample dataset
- X, y = make_regression(n_samples=100, n_features=7, noise=0.1, random_state=42)
- # Split the data
- X_train, X_test, y_train, y_test = train_test_split(X, y, test_size=0.2, random_state=42)
- # Define the parameter grid
- param_grid = {
- 'n_estimators': [100, 200, 300, 400, 500],
- 'max_depth': [10, 20, 30, 40, 50],
- 'max_features': [7],
- 'min_samples_leaf': [1, 2, 4, 6, 8],
- 'random_state': [42]}

# Initialize the RandomForestRegressor

- rf = RandomForestRegressor()

# Initialize GridSearchCV

- grid_search = GridSearchCV(estimator=rf, param_grid=param_grid,

cv=5, n_jobs=-1, verbose=2, scoring='neg_mean_squared_error')

# Fit the grid search to the data

- grid_search.fit(X_train, y_train)

# Extract the best parameters

- best_params = grid_search.best_params_

# Scale the features (optional but can improve performance for some models)

- scaler = StandardScaler()
- X_train = scaler.fit_transform(X_train)
- X_test = scaler.transform(X_test)

# Create random forest regressor

- model = RandomForestRegressor(max_depth=20, max_features=7, min_samples_leaf=2, n_estimators=500, random_state= 42)
- model.fit(X_train, y_train)

# Predict demand

- predictions = model.predict(X_test)
- # Make predictions on training and test sets
- y_train_pred_rf = model.predict(X_train)
- y_test_pred_rf = model.predict(X_test)
- # Calculate RMSE and R² for the training set
- train_rmse = np.sqrt(mean_squared_error(y_train, y_train_pred_rf))
- train_r2 = r2_score(y_train, y_train_pred_rf)

# Calculate RMSE and R² for the test set

- test_rmse = np.sqrt(mean_squared_error(y_test, y_test_pred_rf))
- test_r2 = r2_score(y_test, y_test_pred_rf)
- print(f"Train set RMSE of RF: {train_rmse:.2f}")
- print(f"Train set R² of RF: {train_r2:.2f}")
- print(f"Test set RMSE of RF: {test_rmse:.2f}")
- print(f"Test set R² of RF: {test_r2:.2f}")
- # Plotting the results
- plt.figure(figsize=(14, 6))

# Training data

- plt.subplot(1, 2, 1)
- plt.scatter(y_train, y_train_pred_rf, color='blue')
- plt.plot([y_train.min(), y_train.max()], [y_train.min(), y_train.max()], 'k--', lw=2)
- plt.xlabel('Observed')
- plt.ylabel('Predicted')
- plt.title('Training Data of RF: Observed vs Predicted')

# Test data

- plt.subplot(1, 2, 2)
- plt.scatter(y_test, y_test_pred_rf, color='green')
- plt.plot([y_test.min(), y_test.max()], [y_test.min(), y_test.max()], 'k--', lw=2)
- plt.xlabel('Observed')
- plt.ylabel('Predicted')
- plt.title('Test Data of RF: Observed vs Predicted')print(f"Best parameters found: {best_params}")

:

:

:

**…….**
